# Polyphilicity—An Extension of the Concept of Amphiphilicity in Polymers

**DOI:** 10.3390/polym10090960

**Published:** 2018-08-30

**Authors:** Daniel Heinz, Elkin Amado, Jörg Kressler

**Affiliations:** Department of Chemistry, Martin Luther University Halle-Wittenberg, D-06099 Halle (Saale), Germany; daniel.heinz@chemie.uni-halle.de (D.H.); elkin.amado@chemie.uni-halle.de (E.A.)

**Keywords:** polyphilicity, amphiphilicity, triphilic, self-assembly, hydrophilic, lipophilic, fluorophilic, terpolymers, block copolymers, graft copolymers

## Abstract

Recent developments in synthetic pathways as simple reversible-deactivation radical polymerization (RDRP) techniques and quantitative post-polymerization reactions, most notoriously ‘click’ reactions, leading to segmented copolymers, have broadened the molecular architectures accessible to polymer chemists as a matter of routine. Segments can be blocks, grafted chains, branchings, telechelic end-groups, covalently attached nanoparticles, nanodomains in networks, even sequences of random copolymers, and so on. In this review, we describe the variety of the segmented synthetic copolymers landscape from the point of view of their chemical affinity, or synonymous philicity, in bulk or with their surroundings, such as solvents, permeant gases, and solid surfaces. We focus on recent contributions, current trends, and perspectives regarding polyphilic copolymers, which have, in addition to hydrophilic and lipophilic segments, other philicities, for example, towards solvents, fluorophilic entities, ions, silicones, metals, nanoparticles, and liquid crystalline moieties.

## 1. General Introduction to Philicities/Phobicities

The term *philicity* originates from the Greek language (φίλος-phílos), and means ‘friendly’. In contrast, *phobicity (Φόβος-phobos)* corresponds to ‘fear’. Both terms have been employed frequently in a chemical context, in the sense that philicity is related to attractive forces, and phobicity implies repulsive forces based on the general concept of ‘like dissolves like’. These forces act between different molecules or within compartmentalized molecules. Unfortunately, the terms describing specific philicities/phobicities are commonly used in chemistry, but are frequently not very precise. The possibly best-defined terms from a theoretical and experimental point of view are *nucleophilic* and *electrophilic*, introduced by Ingold in 1933 [1]. Here, a nucleophilic species donates an electron pair during a chemical reaction to an electrophilic species, which results in the formation of a covalent bond. Such interactions between atoms forming covalent or metallic bonds are not considered here. Within this review, we discuss the philicities/phobicities related to the reversible (non-covalent) intra- and intermolecular interactions, which lead to a structure formation by the self-assembly of macromolecules in bulk and in solutions. These intra- and inter-molecular forces result from charges, dipols, and non-polar entities present in macromolecules and their environment. However, all of these interactions are finally electrostatic in nature, that is, they are fully defined by the electron density distribution of the involved (macro)molecules, according to the Hellman–Feynman theorem [2,3]. The combination of philicity and water (ὕδωρ-hýdor) leads to the term *hydrophilic*, probably the first philicity introduced in chemistry. Originally, it was coined for water soluble (macro)molecules. Many polar or ionic molecules (solvents) have attractive physical interactions with water, which result in their water solubility/miscibility. These interactions lack the specificity, stoichiometry, and strong directionality of covalent bonds [4]. Thus, they are ideally suited to keep the molecules in liquids together, without restricting the fast translational and rotational dynamics of these molecules. Typically, 10 to 40 kJ mol^−1^ hydrogen bonds are the strongest physical bonds. Typical intermolecular O–H hydrogen bonds have a length of 0.176 nm. They can also occur between hydrogen and other electronegative atoms in molecules as N, F, or Cl. Therefore, hydrophilic molecules have functional groups susceptible for hydrogen bonding as alcohols, ethers, esters, amines, amides, and so on. It should be noted that not all polar molecules/solvents are hydrophilic [5]. A quantitative measure of the hydrophilicity is the hydration number *nH,* which gives the number of water molecules coordinated per functional group of the molecule. The charged species in water as dissociated salts, acids, or bases, here of course as a function of *pH*-, *pK_a_*-, or *pK_b_*-values, are hydrophilic because of the ion–dipol interactions. It needs to be considered that the entropy of mixing ∆*S_m_* with water can be negative, caused by the structuring of water, which can counterbalance, to a certain degree, the exothermal contributions of the enthalpy of mixing ∆*H_m_* to the Gibbs free energy of mixing ∆*G_m_*. Functional groups alone are not sufficient to understand the hydrophilic character of molecules, as all parts of the molecule need to be considered. Thus, in the homologous series of alcohols (from methanol, ethanol, to fatty alcohols) or carboxylic acids, starting from formic acid and acetic acid towards fatty acids, as palmitic acid or stearic acid, the hydrophilicity is decreasing. This can be explained with the concept of the hydrophobic effect, originally developed by Tanford, for proteins [6]. They are formed from 20 native amino acids, which are hydrophilic or hydrophobic, depending on their remaining functional group after incorporation into proteins via peptide bonds. Thus, their structure formation and functionality are not only governed by their hydrophilic amino acids, but also by their hydrophobic amino acids, resulting in repulsive interactions with water. This concept was also extended, for example, to the homologous series of monohydroxy alcohols [7]. Here, the OH-group is able to form hydrogen bonds to water, but the hydrophobic parts of the alcohols influence several other physical properties, such as dielectric relaxation, solvation dynamics, and so on. Thus, the term *hydrophobic* is usually considered as the opposite of hydrophilic. The synonymous use of *oleophilic* for lipophilic and *oleophobic* for hydrophilic should be mentioned here. There are several concepts to quantify the hydrophilicity/hydrophobicity of molecules, as will be discussed below.

This review deals with the concept of hydrophilic and lipophilic macromolecules. When both of the properties are combined within one polymer, *amphiphilic* macromolecules are formed. As most perfluorinated species are neither hydrophilic nor lipophilic, we first extent this concept to *triphilic* molecules. Caused by the fact that a plethora of special philicities do exist, we finally introduce the general term *polyphilicity* of macromolecules. The incorporation of several different philicities in a single polymer multi-block architecture leads to unprecedented possibilities of structure formation by self-assembly on the nano- and mesoscales, and thus it opens new roads to sophisticated applications of the polymers in enabling technologies.

## 2. Quantitative Approaches to Philicities

### 2.1. Solubility Parameter Concept

As the name ‘solubility parameter’ indicates, this concept was initially developed to judge the solvent quality for the dissolution of different species, for example, polar or non-polar ones. A starting point for the quantitative discussion is ∆*U_v_*, the molar internal energy of vaporization. Its negative value, −∆*U_c_*, is the molar cohesive energy, that is, the energy related to the net attractive interactions that keep the molecules together in the condensed state. The −∆*U_c_* divided by the molar volume, *V*, is called the cohesive energy density, *c*, Equation (1):

(1) $$c=\left(-∆U_{c}/V\right)$$

This term was then employed by Hildebrandt and Scott [8,9,10], to define the solubility parameter *δ*, Equation (2):

(2) $$\delta =c^{0.5}={\left(-∆U_{c}/V\right)}^{0.5}$$

The dimension of the solubility parameter is thus given in (J∙m^−3^)^0.5^ or (MPa)^0.5^. There have been several refinements of the solubility parameter approach, for example, by Hansen et al., separating the solubility parameter into dispersive, polar, and hydrogen bonding terms [11,12,13]. For solvents, the solubility parameter can directly be obtained from experiments determining the enthalpy of evaporation. For very polar solvents with a strong hydrogen bonding tendency, the solubility parameters range from above 30 MPa^0.5^ such as, for example, ammonia, ethylene carbonate, glycerol, and formamide, up to the highest value for water of 47.9 MPa^0.5^ [14]. So, the polar species with several possibilities for intermolecular interactions have a large solubility parameter, as it needs more energy to overcome them during the evaporation process. In contrast, the small solubility parameters are obtained, for example, for the alkanes or fluorinated species in the range of 11 to 15 MPa^0.5^ [14]. The scaling of the internal energy of evaporation with the molar volume is also easy to understand when comparing, for example, the homologous series of the *n*-alkanes. They all have only weak intermolecular van der Waals interactions in the range of ~1 kJ·mol^−1^. Nevertheless, methane is a gas at room temperature and normal pressure, *n*-butane is a liquid, and *n*-decane is a solid, as a result of the increasing molar volumes.

Caused by the negligible vapor pressure of polymers, it is impossible to measure their enthalpy of evaporation, and thus their solubility parameters, directly. Several other physical quantities can be related to the enthalpy of evaporation and thus to the solubility parameters [15]. The solubility parameters can directly be obtained from pressure, volume, and temperature (PVT) data, using the relation derived by Olabisi and Simha [16], Equation (3).

(3) $$\delta^{2}\approx T\alpha /\kappa$$

where T is the absolute temperature, *α* is the thermal expansion coefficient, and *κ* is the compressibility. Frequently group contribution methods have been employed to calculate the solubility parameters of the polymers as a rather rough estimation [17,18,19].

In order to judge the solubility of polymer (component 1) in a certain solvent (component 2), the interaction parameter *χ*_1,2_ is introduced. It is given within the framework of regular solutions as (Equation (4)):

(4) $$\chi_{1,2}=V_{seg}{\left(\delta_{1}-\delta_{2}\right)}^{2}/RT$$

where *V_seg_* is the volume of a polymer segment. In this theory, specific interactions are excluded, as *χ*_1,2_ is always positive. The smaller the difference between the two solubility parameters, the smaller *χ*_1,2_ is. Thus, the solubility of the polymer in the solvent is given, as long as the positive enthalpic contribution to the Gibbs free energy of mixing is smaller than that of the combinatorial entropy of the mixing given in the framework of the Flory–Huggins theory [20].

### 2.2. The Concept of Hydrophilic–Lipophilic Balance (HLB)

The original concept of the hydrophilic–lipophilic balance (HLB) was developed by Griffin [21]. He investigated emulsifiers containing hydrophilic and lipophilic groups. Thus, numerical values of 1 were given to pure oleic acid, and 20 to sodium oleate, indicating that the sodium salt is the better emulsifier. Later, Griffin introduced the HLB values for certain non-ionic surfactants having hydrophilic and lipophilic groups. It was intended to provide a practical guide for the application of amphiphilic molecules (mainly surfactants) in pharmaceutical and cosmetic industry [22]. Examples for this approach are alkyl ether and esters of poly(ethylene oxide) (PEO). Here, the HLB value (Equation (5)) is given by:

(5) HLB=E/5

where *E* is the mass percentage of the PEO in the molecule. For esters of multivalent alcohols with fatty acids, the HLB value is defined as follows (Equation (6)):

(6) $$HLB=20\;\left(1-\frac{S}{A}\right)$$

where *S* is the saponification value of the ester and *A* is the acid value of the respective fatty acid. For polymer chains containing poly(ethylene oxide) and a polyhydric alcohols (as glycerol or sorbitol), the following Equation (7) was proposed:

(7) $$HLB=\frac{E+P}{5}$$

where *P* is the mass percentage of polyhydric alcohol content.

A general expression for the calculation of the HLB values of amphiphilic (macro)molecules is given by Griffin [22], as (Equation (8)):

(8) $$HLB=20\left(\frac{M_{h}}{M}\right)$$

here, *M_h_* is the molar mass of the hydrophilic portion and *M* is the molar mass of the whole macromolecule. Thus, a polymer with an HLB value close to 0 is completely lipophilic/hydrophobic, and with a value close to 20 it is completely hydrophilic/lipophobic. As a rough estimation, the macro(molecules) with HLB values in the range between 6 and 13 are generally amphiphilic materials forming micelles in aqueous solutions [23].

Davis developed an advanced *HLB* approach [24], (Equation (9)), as follows:

(9) HLB=7+ΣA−ΣB

where *A* and *B* represent the hydrophilic and hydrophobic group constants contributions, respectively [25,26].

### 2.3. Concept of Partition Coefficients

The concept of partition coefficients is based on Nernst’s distribution law, which defines the ratio of a solute distributed between two immiscible solvents. Using polar solvents such as water or alcohols on the one hand, and non-polar solvents such as cyclohexane or *n*-octane on the other hand, allows for the determination of the hydrophilic or lipophilic character of the solute. The most prominent partition coefficient *log P_O_*_/*W*_ (Equation (10)) considers the distribution of molecules between 1-octanol and water [27,28,29], calculated as follows:

(10) $$logP_{O/W}=log\left(\frac{{\left[solute\right]}_{octanol}}{{\left[solute\right]}_{water}}\right)$$

It describes the portioning of biologically and pharmaceutically relevant molecules between biophases (e.g., proteins, DNA, and biocompatible polymers) enriched in 1-octanol or water. An outstanding correlation has been found between the 1-octanol/water partition coefficients and the biological activity of drugs, which is better than for the oil/water or alkane/water partition coefficients. For example, the 1-octanol/water partition coefficients of gaseous anesthetics were used to calculate their lipophilicity, defined by Hansch [30] as *log P_O_*_/*W*_ and a polar factor. It correlates well with the anesthetic potency of the gases. As the polar functional groups with hydrogen atoms also influenced the anesthetic effect, a structure-activity correlation was formulated based on polarity and lipophilicity [31]. A detailed investigation of the 1-octanol/water partition coefficients for 600 organic compounds was carried out by Sangster [28]. The main factors influencing the 1-octanol/water partition are the size of the solute, the solute polarizability, and the hydrogen-bond basicity. The solutes able to accept hydrogen-bonds or being polarizable partition preferentially in the aqueous phase, while the solutes of an increasing size show preference for the 1-octanol phase [27]. As 1-octanol possesses a lipophilic tail and a polar headgroup, it is amphiphilic and resembles the proteins and phospholipids present in the cell membranes, which makes it suitable as a model for investigating the partitioning in such biological systems [29]. In the case of using fluorinated species, a partition coefficient between perfluoro(methylcyclohexane) (PFMC) and toluol (*ln P*_PFMC/Tol_) is useful, especially when using fluorophilic fluorescence dyes for their determination [32,33].

Frequently, the partition coefficients of a solute between polymer and a liquid (which is not a solvent for the polymer) have been determined [34]. These partition coefficients are extremely important for processes such as extraction [35,36] and fractionation [37,38]. Most of the measurements are carried out for the partitioning of organic molecules between hydrophobic polymers and water [39].

### 2.4. Polarity Values

The term ‘polarity of a molecule’ is still not clearly defined [40,41]. It can be interpreted in many different ways and can be measured by several different methods. *Polarity* can be considered as (i) the sum of all of the molecular interactions between the molecule and the solvent, (ii) the permanent dipole moment of the molecule, or (iii) the relative electric permittivity of the molecule [40,42]. The dielectric constant of a solvent is often used to determine its polarity quantitatively. However, this approach results in solvents being considered as an isotropic, unstructured set of molecules that do not interact. The dipole moment used in this theory is also only partly suitable for the definition of polarity, as the charge distribution in a molecule depends on many more factors than just the dipole moment (e.g., quadrupole or multipole moments) [43]. The many different views on the polarity of a molecule are discussed by Katritzky et al. [41].

The first polarity measurements were carried out in 1948 by Grunwald and Winstein (*Y*-value) [43]. One of the most important methods is the determination of polarity by UV spectroscopic measurements. The *E_T_* value and $E_{T}^{N}$ value are of huge significance (Equation (11)).

(11) $$E_{T}=h\cdot c\cdot \tilde{v}\cdot N_{A}=\left(2.859\cdot 10^{-3}\cdot \tilde{v}\right)\;in\;kcal\cdot mol^{-1}\;\mathrm{or}\;\left(1.196\cdot 10^{-2}\cdot \tilde{v}\right)\;in\;kJ\cdot mol^{-1}$$

where *E_T_* stands for the molar electronic transition energy or molar electronic excitation energy in kJ·mol^−1^ or kcal·mol^−1^, *h* is Planck’s constant, *c* is the speed of light, $\tilde{v}$ is the wavenumber, and *N_A_* is the Avogadro number.

Investigations carried out in the 1940s on the shifting the absorption maxima of various dyes [44,45] led to the realization that the absorption maxima shift depended on the polarity of the solvents used. *N*-ethyl-4-carbomethoxy pyridinium iodide was used as the first test dye for the determination of a polarity scale of various solvents [46,47,48]. Increasing the solvent polarity leads to a blue shift (hypsochromic, negative solvatochromism) and a reduction in polarity to a redshift (bathochromic, positive solvatochromism) [41]. The determination of the polarity of strongly polar or water-containing solvents is not possible with the dye mentioned above [49]. Thus, other dyes were used to solve this issue [49]. The dye derivatives that were used by Dimroth and Reichardt and were named after them, with the basic structure, 2,6-diphenyl-4-(2,4,6-triphenylpyridinio)phenolate, were used to determine the *E_T_* value of a large number of molecules. The empirical value *E_T_*(30) (in kcal·mol^−1^, Equation (12)) is defined as the molar transition energy of the intramolecular charge transfer absorption ($\lambda_{max}$ in nm) of the Dimroth–Reichardt dye (diphenyl betain 30 [49]) used, at 25 °C and a pressure of 0.1 MPa [40,41].

(12) $$E_{T}\left(30\right)=28591/\lambda_{max}$$

For the calculation of the normalized dimensionless $E_{T}^{N}$-value, the following Equation (13) is used (TMS—tetramethylsilane):

(13) $$E_{T}^{N}\left(solvent\right)=\frac{E_{T}\left(solvent\right)-E_{T}\left(TMS\right)}{E_{T}\left(water\right)-E_{T}\left(TMS\right)}$$

A summary of the *E_T_*(30)- and $E_{T}^{N}$-values for solvents can be found elsewhere [40]. For example, the *E_T_*(30) value for water is 63.1 kcal·mol^−1^, for methanol 55.4 kcal·mol^−1^, and for dimethyl sulfoxide 45.1 kcal·mol^−1^. The polarity values of the fluorinated solvents can be determined using the fluorinated dyes developed by Middelton [50].

Attempts were also made to correlate the polarity values measured by different analytical methods. It should be noted that many solvent polarity scales correspond to each other and can be divided into approximately the following five large solvent groups: (a) intramolecular hydrogen bonds; (b) strongly protic, strong hydrogen bonds; (c) dipolar, aprotic, strong hydrogen acceptors; (d) esters, ethers, amines, and alkyl halogens, whose polarity is not smaller than that of groups (b) and (c), but also not much larger than that of groups (d); and (e) apolar [41]. By taking the *K* coefficient of the Mark–Houwink–Sakurada equation as a function of the two polarity parameters of the solvent, for example, *E_T_*(30) and π* (polarizability), a polarity parameter for the polymers can be derived [51]. 

It is important to realize that a high polarity value does not necessarily mean that the polymer is also hydrophilic. Poly(acrylonitrile) is, for example, highly polar, but it is strongly hydrophobic. As the CN-group is not protic, it cannot develop specific interactions with water. Thus, another important quantity in order to judge the hydrophilicity, the hydration number, has been employed. The more hydrophilic the polymer, the more water it can bound, which results in a higher hydration number [52,53]. 

## 3. Philicities/Phobicities in Polymeric Systems

**Figure 1:**
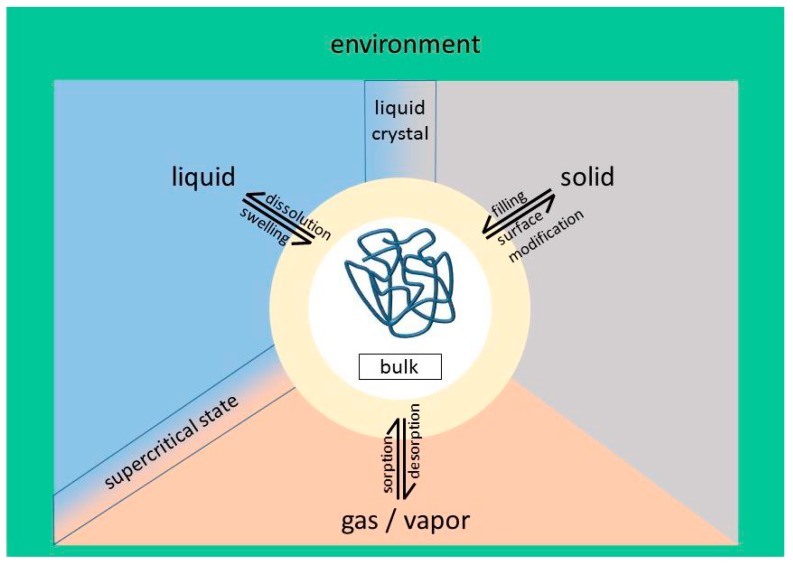
Schematic representation of possible interactions of polymers with their environment.

Figure 1 shows different possibilities for interactions of polymeric systems. The starting point is the bulk polymer. This can be homogeneous, for example, as an amorphous solid or melt. The bulk phase can be heterogeneous such as a semi-crystalline homopolymer, as it consists of a crystalline and an amorphous phase. The polymers can also form several thermotropic liquid crystalline phases in bulk. The microphase separated block copolymers [54,55] or graft copolymers [56] can also be considered. Typically, such bulk phases are not discussed in terms of philicities. The phase separation is well understood in terms of classical thermodynamics and statistical mechanics [57,58]. Philicities/phobicities are employed when considering the polymer–environment interactions, as shown in Figure 1. Polymers can interact with liquids, solids, and gases.

Regarding the polymer-liquid (solvent) interactions, frequently used terms for macromolecules are also hydrophilic and lipophilic (synonymous with oleophilic). They usually stand for non-covalent interactions with surrounding solvents. The term hydrophilic indicates polar polymers that are water soluble or absorb water connected with swelling, or at least have strong interactions with water, such as the formation of hydrogen bonds. A general term to indicate the favorable interaction between a polymer and a solvent is *solvophilicity*. This term is frequently employed for the self-assembly of amphiphilic block copolymers in selective solvents [59]. Also, polyelectrolytes as poly(methacrylic acid) or poly(vinyl amine) are considered to be hydrophilic, nevertheless, the water solubility strongly depends on the *pH*-value or salt content of the aqueous solution. In contrast, hydrophobic means attractive interactions of polymers with non-polar solvents, such as alkanes, benzene, or triglycerides. Of course, there are gradual differences between hydrophilic and hydrophobic solvents, as can be easily imagined for tri-, di-, and mono-glycerides, or identical glycerides with different chain lengths of the fatty acids. Polymers can also be dissolved in liquid crystalline solvents and form lyotropic phases [60]. Also, stiff polymers can be dissolved and form lyotropic liquid crystalline phases, with the most famous example being Kevlar^®^ dissolved in sulfuric acid [61]. Of course, polymer networks cannot be dissolved, but can only be swollen to different degrees. The swelling of polymer networks is a standard procedure to determine the polymer solubility parameters experimentally [62,63]. Poly(vinyl alcohol) is usually considered as water soluble and can also form physical polymer networks after freeze–thawing cycles of its aqueous solutions. Because of polymer crystallization, stable hydrogels are formed [64,65].

As for interactions between the polymers and surrounding gas phases, most investigations have focused on gas-responsive polymers, that is, the possibility of triggering specific changes in polymer conformation, or aggregation states, through the interaction of functionalities on the polymer chains and a gas [66]. The key gases investigated up to now have been oxygen (O_2_), nitrogen (N_2_), carbon oxides (CO or CO_2_), nitrogen oxides (NO*_x_*), sulphur oxides (SO*_x_*), and hydrogen sulfide (H_2_S), mainly because of their relevance in environmental and health issues [67,68,69].

Supercritical CO_2_, which is neither a gas nor a liquid, has a tremendous amount of applications in polymer technology. It is used as non-polar solvent for several polymers, for example, for poly(acrylates), including methyl, ethyl, propyl, butyl, ethylhexyl, and octadecyl; for poly(butyl methacrylate); for poly(vinyl acetate); and for fluorinated random copolymers. However, it cannot dissolve polyethylene, poly(acrylic acid), poly(methyl methacrylate), poly(ethyl methacrylate), polystyrene, poly(vinyl fluoride), or poly(vinylidene fluoride) [70,71,72]. It is also applied as a processing aid in polymer extrusion [73]. The term CO_2_-philicity has been introduced for polymers that are soluble in supercritical CO_2_ [74,75].

Furthermore, polymers can also interact with solid surfaces in applications such as filled polymers [76], nanocomposites [77], or coatings [78]. In addition, depending on the surface chemistry of the solid surface, in this case, hydrophilic, lipophilic, and fluorophilic interactions with the respective polymer can be present.

## 4. From Polymer Amphiphilicity to Polyphilicity

**Figure 2:**
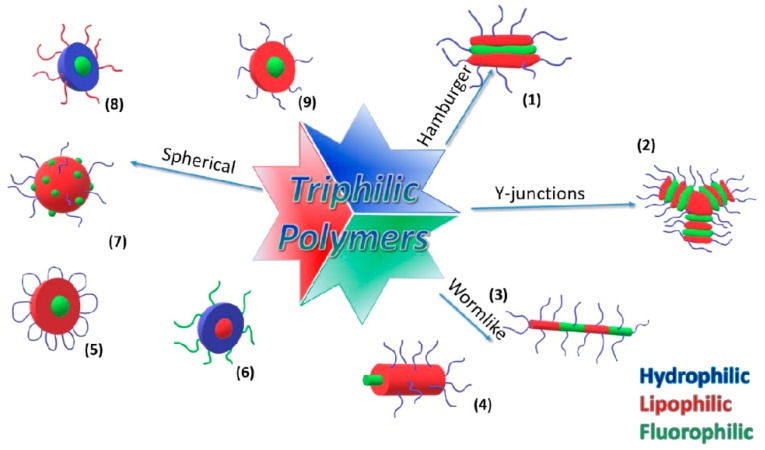
The diversity of possible micellar aggregates of triphilic polymers in aqueous solution. (1)—Hamburger; (2)—Y-junctions; (3)—segmented wormlike; (4)—wormlike; (5)—flower-like; (6)—lipophilic core, hydrophilic-fluorophilic corona; (7)—raspberry-like; (8)—fluorinated core; hydrophilic-lipophilic corona; (9)—fluorinated core, lipophilic-hydrophilic corona.

An attempt of the classification from a chemical standpoint of the wide variety of polymers that can be synthesized via the different polymerization techniques and post-polymerization reactions is based on the number of different *chemical families* (e.g., methacrylates, acrylates, styrenics, etc.) present in a given polymer structure [79]. Within that frame, three chemically distinct block types joined in any imaginable architecture (linear ABC or ABCBA, miktoarm star µ-ABC, or a closed loop) are termed ‘terpolymers’, with four distinct block types forming a tetrapolymer, five block types forming a pentapolymer, and so on. Another attempt of classification considers not the number of chemical families, but the number of distinct *chemical affinities* present in the polymer. The affinity could be directed towards a solvent, a surface, an interface, nanoparticles, or the surrounding medium. A polymer with three chemically distinct blocks, two of them hydrophobic and one hydrophilic (as e.g., in poly(ethylene oxide)-*b*-poly(styrene)-*b*-poly(isoprene)), is called *amphiphilic*. If an additional fluorophilic block is included, the resulting polymer would be *triphilic*, and an additional block with, for example, a gold affinity via an SH-group, would render a *tetraphilic* polymer. In general, a polymer with multiple chemically distinct blocks is called *polyphilic*. The differences between both of the classification schemes are not only semantic and become more evident when a block copolymer is surrounded or in the near proximity of a medium/interface selective for only one of the blocks, which triggers the formation of self-organized structures. For example, a linear ABC triphilic polymer in a good solvent for only one of the blocks would self-assemble in a rich variety of microphase separated morphologies (e.g., core-shell micelles or multicompartment micelles). In contrast, a linear ABC terpolymer in this situation might form simple micelles or flower-like micelles instead (see illustrations in Figure 2). In this review, we follow the classification based on chemical affinities, starting from the simple ones.

### 4.1. Hydrophilic, Lipophilic, and Amphiphilic Polymers 

The most common chemical affinities normally found in polymers are ‘lipophilicity’ (i.e., affinity towards organic non-polar solvents, such as hydrocarbons), and ‘hydrophilicity’ (i.e., affinity to water or other polar solvents). The concept of amphiphilic molecules was successfully extended to polymers by the anionic synthesis of the AB diblock copolymers, where one block is hydrophilic and the other is lipophilic [80]. Of course, most of the synthetic polymers are hydrophobic (e.g., polyolefins, most polyacrylates, most polymethacrylates, polyimides, polycarbonates, etc.). However, mixed affinities are also observed, such as in polyamides and polyesters. In such polymers, although the functional linkage groups of the polymer backbone themselves are hydrophilic, and are able to form hydrogen bonds, these polymers are usually not water soluble, because of the presence of hydrophobic alkyl or phenyl groups between the linkage groups. As an important result, these polymers show a certain extent of water uptake, which strongly influences their physical properties.

Classical synthetic water soluble and thus hydrophilic polymers contain several polar functional groups, such as poly(vinyl alcohol), poly(acryl amides), poly(methacryl amides), or poly(vinyl methyl ether) (PVME). Another important hydrophilic and water soluble polymer is PEO. Its water solubility is rendered by the polar ether bond and the shortness of the hydrophobic two methylene groups. Increasing the number of hydrophobic groups, e.g., in poly(propylene oxide) (PPO) or poly(tetrahydrofuran), already leads to water insolubility. Nevertheless, PPO remains weakly hydrophilic and is frequently employed in block copolymers with PEO. A less known fact is the amphiphilic character of PEO. After spreading PEO from a chloroform solution onto the water surface of a Langmuir trough, it remains there and does not submerge into the water subphase [81]. Responsible for this behavior are the two hydrophobic methylene groups per monomer unit. The peculiarities of the PEO and PPO behavior in water illustrate the point that hydrophilicity is always the result of several interacting structural factors. Thus, there are different degrees of water affinity, even among perfectly water soluble polymers. A surprising consequence is the self-assembly of completely water-soluble double hydrophilic block copolymers (DHBCs) into organized structures in aqueous medium [82], for example, the giant vesicles formed by PEO-*b*-polysaccharides in water [83]. Such DHBCs are not considered amphiphilic in the classical sense, as the phase separation process is not driven by a solvent selective for one particular block, but rather by a slight difference in water affinity.

A large group of hydrophilic polymers belong to the class of polyelectrolytes, where the water solubility, and thus the hydrophilicity, is *pH*-dependent. For instance, poly(acrylic acid) or poly(methacrylic acid) are usually referred to as water soluble, but in fact, these polymers are only soluble as salts at *pH*-values above their *pK_a_*-value. 

### 4.2. Triphilic Polymers—Hydrophilic, Lipophilic, and Fluorophilic

The linkage of fluorinated segments with hydrophilic or lipophilic segments [84,85,86,87,88,89,90,91] creates a new class of amphiphilic polymers, different from the typical hydrophilic-lipophilic ones, as the fluorinated segments are incompatible with both lipo- and hydrophilic segments, and at the same time, provide interesting and useful properties [92,93]. Particularly, the unusual behavior of fluorinated compounds in biological systems, including their increased metabolic stability, an enhanced permeation through the cell membranes or a stronger binding affinity to specific protein sequences, is currently attracting much attention in biomedical research, and has been the topic of a recent review [94]. Furthermore, when a hydrophilic–lipophilic polymer is supplied with a third structural element, it expands the possibilities for the development of self-assembled multi-compartment macromolecules [92,93,94,95,96,97,98,99,100,101,102,103,104]. It often has remarkable effects on the catalytic behavior [92,105,106], the interaction with biological membranes [107,108,109,110], or the transport of active substances [103,109,111,112].

**Figure 3:**
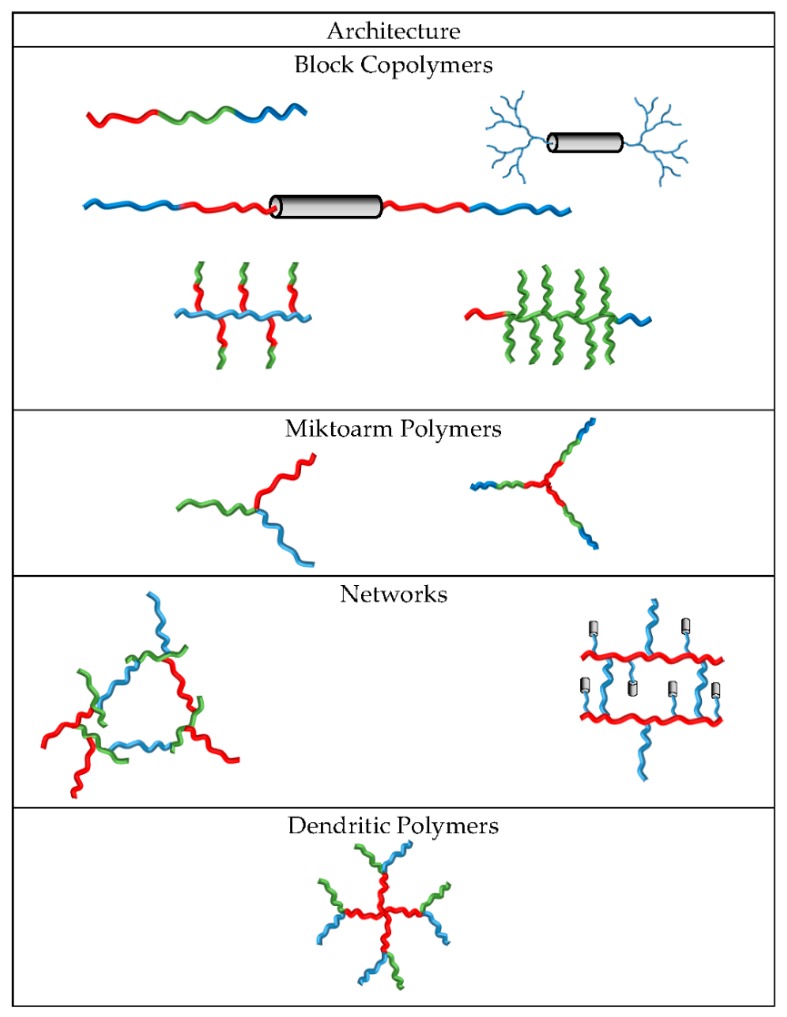
Examples for triphilic polymer architectures.

One of the first attempts to combine fluorophilic, hydrophilic, and lipophilic segments into one polymer was done by the group of Lodge. They synthesized by anionic polymerization a linear triblock copolymer (poly(ethylene oxide)-*b*-poly(styrene)-*b*-1,2-poly(butadiene)), in which the butadiene block was selectively modified with *n*-perfluorohexyliodide [96]. They also linked a perfluorinated chain (poly(perfluoropropylene oxide)-PFPO) with a PEO and a poly(ethylethylene) chain (PEE) [113]. The term triphilic, for polymers with hydrophilic, lipophilic, and fluorophilic segments, was first used by Krafft in 2007 [114]. It was based on a report published in 2004 on the behaviour of ABC miktoarm star polymers (Figure 3) in water [115]. The term triphilic has also been used in other contexts in different fields, for example, in the separation by flotation with triphilic collector molecules [116,117]. Liquid crystals consisting of two flexible side chains (alkyl and fluorinated chain) and a fixed biaryl core were also referred to as triphilic [118]. There is a great variety of polymer architectures accessible by arranging three different segments ((A) hydrophilic, (B) lipophilic, and (C) fluorophilic) in a macromolecule (Figure 3). The polymers with the arrangement of ABC [95,119,120,121], BCA [103,122], and CABAC [123,124], should be mentioned, whereby the fluorinated part can be located in the main chain [103,111,124,125,126,127,128] or in the side chain [95,120,121,129,130]. These polymers have been investigated in many ways, particularly regarding their behavior in aqueous environments. For instance, triphilic polymers with the structure ACB form nanoparticles that contain a hydrophobic core and can therefore be used as vehicles for the transport of hydrophobic drugs [103]. When the fluorinated and lipophilic components change positions, polymers with the structure ABC are obtained. For such polymers, spherical micelles with fluorine-rich domains are formed and can be visualized by cryo-electron microscopy [121]. Such compartibilized micelles are the result of superstrong segregation. This means that the interaction between the different blocks is so strong that the interfacial interaction overcomes the entropic barrier and thus prefers the formation of flat interfaces/structures. The shortest blocks, in this case the fluorinated part, are arranged fully extended next to each other [101,131,132]. The formation of spherical micelles having a fluorinated core, lipophilic intermediate shell, and a hydrophilic corona (Figure 2(**9**)) [133] or **networks** (cyclic structures, see Figure 3) with ABC triblock polymers, are also possible [97,98,134,135]. The polymers with the general structure CAB dissolved in water have been also investigated. In the case of the block copolymer of C_9_F_19_–PGMA*_z_*–PPO_34_ (perfluorinated moiety–poly(glycerol methacrylate)–poly(propylene oxide)) micelles with a fluorinated core and PPO were formed as corona enhancers (Figure 2(**8**)) [125]. The polymers with the structure CABAC were synthesized with different block lengths. The variable hydrophilic part is formed by the poly(glycerol methacrylate) (PGMA) block. The lipophilic poly(propylene oxide) and the fluorinated part (C_9_F_19_-) had the same length in both of the polymers. For these polymers, a critical micelle concentration (CMC) of 9.5 µM was found for the polymer with the shorter hydrophilic block (PGMA_24_), and 2.5 µM for the polymer with the longer one (PGMA_42_). Dynamic light scattering experiments showed that the formation of a species with a smaller hydrodynamic radius occurs when the critical micelle concentration (CMC) is exceeded. Further investigations by NMR and transmission electron microscopy (TEM) led to the model proposed in Figure 2(**5**). Longer hydrophilic segments allow the chain to fold back and form flower-like micelles, in which the core forms the fluorinated unit (Figure 2(**6**)). In the case of the shorter hydrophilic block, however, such backfolding does not occur and micelles with a lipophilic core and the fluorinated part of the polymer are detected in an aqueous environment [136]. Monolayer investigations of the shorter polymer with (PGMA_24_) at the air–water interface and analysis of the interaction of lipid monolayers with the triphilic and amphiphilic (without fluorinated part) block copolymers were performed. It is concluded that the fluorinated segments located at the polymer end in the triphilic polymer significantly inhibit its removal from the lipid layer, compared to amphiphilic molecules [108]. 

Another possible variation are **star** polymers [100,111,113,137,138,139]. The length of the individual arms can be varied in many ways, and their behavior in an aqueous system was investigated. A ternary phase diagram shows the influence of the individual chain lengths on the micelles or aggregated structures [137]. For the polymers with a low volume fraction of PFPO in the micelle core, for example, the formation of segmented wormlike bands (Figure 2(**3**)), Y-junctions (Figure 2(**2**)) or networks is preferred. Wormlike multicomponent micelles (Figure 2(**4**)) or raspberry-like micelles (Figure 2(**7**)) can be formed when the fluorinated part increases compared to the lipophilic part [137]. In the case of very long hydrophilic fractions, Hamburger structures may form where the fluorinated segment has very strong hydrophobicity and is shielded from lipophilic segments at the top and bottom (Figure 2(**1**)) [92,137].

**Dentritic** polymers (Figure 3) are highly complex structures. The starting point of such a synthesis is a molecule with several modifiable groups. The polymer grows from this starting molecule and branches out again with each subsequent step [138,140,141,142]. Like the structure of triphilic polymers, the synthesis routes to these structures are also multifaceted. In the following, some of these various possibilities will be described in more detail. One possibility is anionic polymerization [104,113]. 

Using this method, Lodge et al. synthesized µ-ABC star triblock polymers. Starting with a lithium-modified initiator, trans-butadiene was polymerized in THF at −60 °C, the remaining double bonds in the poly(butadiene) were reduced, and, in the next step, oxirane was added and linked together with the fluorinated chain [113]. 

A greater synthetic importance, other than anionic polymerization, has been reached by atom transfer radical polymerization (ATRP) [90,104,141,143,144] and reversible addition-fragmentation chain transfer (RAFT) polymerization [119,138,145,146,147]. Both methods belong to the large group of reversible-deactivation radical polymerization (RDRP) techniques. An advantage of ATRP is the precise determination of the polymer end groups. The basic structure of the initiator for ATRP possesses a labile C–Cl or C–Br bond at the end, which can initiate polymerization after homolytic cleavage and can later be exchanged by other functional groups [148]. Ligands, solvents, and monomers can be selected in a wide range [149,150,151]. For example, dendrimers with fluorinated segments were produced using several sequential ATRPs [141]. The polymerization of solketal methacrylate, following removal of the solketal protective groups led to the synthesis of poly(glycerol methacrylate). The initiator can be, for example a low molar mass fluorinated chain, or a poly(propylene oxide) as macroinitiator [90,94,124]. A major advantage is the interchangeability of the halogen end group with another functional group. In some cases, the halogen is replaced by an azide group [143,152]. After polymerization with solketal methacrylate, the fluorinated end groups could be added to the macroinitiator, mentioned before, with the aid of copper(I)-catalyzed alkyne-azide cycloaddition (CuAAC) [124]. With the help of CuAAC, it is possible to create complex structures, such as conjugates of proteins and polymers [153], through relatively simple reactions [154,155,156,157,158]. It can be concluded that triphilic block copolymers, with their large variety of architectures and chemical structures, open the door to a wide range of sophisticated applications. 

### 4.3. Additional Motives and Polyphilicity

**Figure 4:**
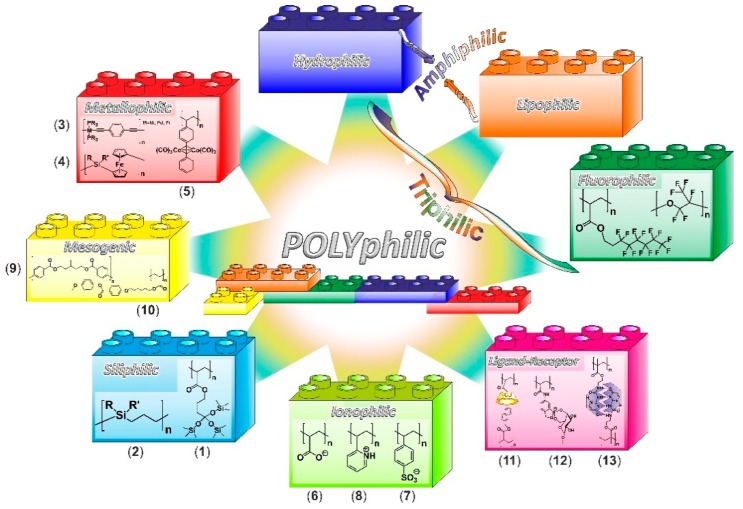
Representative structure motifs present in polyphilic polymers. (1,2)—Siliphilic; (3–5)—metallophilic; (6–8)—ionophilic; (9,10)—mesogenic; (11–13)—ligand-receptor.

Besides the already discussed philicities (hydrophilicity, lipophilicity, and fluorophilicity) most often found in polymer science, there is a wide variety of additional structure motifs that have been more recently included in multi-block copolymers, in order to confer a particular affinity to the polymer. The different compatibilities present within the same polymeric structure trigger its self-assembly in bulk or in selective solvents, generating nanoscale domains of distinctly chemical affinities. Representative examples of structure motifs are presented in Table 1, and the most popular chemistries for each group are shown in Figure 4.

#### 4.3.1. Siliphilic Segments

Siloxane blocks (Figure 4(**1**)) form amorphous soft domains, with low transition temperatures and good chemical stability. Being amorphous, they exhibit higher gas permeability and higher solubilities of hydrophobic solutes than crystalline domains, which makes them interesting for micro-compartmentalization applications [159]. On the other hand, carbosilane segments (Figure 4(**2**)) containing silicon atoms in the main chain, form crystalline domains if substituted with methyl groups, and highly flexible amorphous domains if longer substituents are present. They exhibit a particular affinity for glass interfaces [160,161].

#### 4.3.2. Metallophilic Segments

The term metallophilic is normally employed in biology to describe proteins (i.e., biological polymers) with a high affinity for metal ions (e.g., lactoferrin, transferrin, or ferroportin). Recently, such proteins have been conjugated to polymersomes [162] and dendrimers [163], creating metallophilic drug or gene delivery systems. Organometallic blocks containing metal centers can be viewed as polymer–metal hybrids on a molecular level. They are prone to metal–metal interactions and can generate domains with distinctly and interesting electronic, optical, and magnetic properties [164]. Alternatively, the metal-rich domains can be used as patternable precursors of metal nanoclusters, with promising applications as templates in nanolithography or as catalysts [165]. The metal centers typically belong to the d-block transition metals, although p- or f-block metals are also possible, and can be located either along the main chain or in the side groups. 

Among the most intensively investigated main-chain metalloblocks are polymetallaynes (Figure 4(**3**)) and polyferrocenylsilanes (Figure 4(**4**)). Polymetallaynes are conjugated polymers with metal atoms linked through acetylenic units. Such segments are rigid-rods, and the inclusion of electron-rich metal centers into a *π*-conjugated backbone confers them functional properties, such as photoluminescence, redox activity, and optoelectronic features [166]. Poly(ferrocenylsilane)s are synthesized by ring-opening polymerization of silicon-bridged ferrocenophanes. They possess interacting iron atoms along the backbone. In selective solvents, segments of poly(ferrocenyldimethylsilane) (Figure 4(**4**), R = R’ = Me) possess a crystalline nature and are prone to form unusual micrometer length cylindrical micelles (iron-rich nanorods) [167,168]. Additionally, the iron-rich domains formed by poly(ferrocenylsilane) segments can be thermally (pyrolysis) or UV-ozone converted into iron nanoclusters, useful for particular applications, such as nanolithography or as heterogeneous catalysts for the growth of carbon nanotubes [169]. A different type of main-chain metalloblocks are those containing gold (I) centers, which are obtained from the condensation of diphenylphosphanylbenzoic acids [170]. The resulting gold-carboxylate motifs along the polymer backbone have the tendency to form secondary *aurophilic* bonds that can be used to direct self-assembly. Remarkable double-stranded helical structures are obtained through such aurophilic bonding.

Side-chain metalloblocks incorporate transition metal complexes into groups pendent to a non-conjugated main chain. An illustrative example are the blocks incorporating cobalt hexacarbonyl motifs (Figure 4(**5**)), which form cobalt-rich domains through self-assembly. They are useful as precursors of cobalt magnetic nanoparticles, obtainable after mild heating [171]. Also interesting are the iridium phenylpyridine moieties that are suitable as luminescent probes, because of the luminescence of the iridium (III) complexes [172].

In contrast to the covalently bonded metal centers considered above, another type of metallophilic polymer incorporates metal centers linked through reversible coordinative or metallophilic interactions, so called coordination or supramolecular polymers. The reversibility of the binding renders the polymers extremely dynamic in nature. Selected examples are one-dimensional silver-based polymers held together through Ag···Ag *argentophilic* interactions [173], [174], and Au(I)-thiolate coordination polymers, including Au···Au aurophilic interactions [175].

#### 4.3.3. Ionophilic Segments

Blocks incorporating charged motifs have an affinity to participate in self-assembly processes through electrostatic interactions either with themselves, with inorganic/organic divalent or multivalent counter ions, or with oppositely charged polymeric segments. For example, polyanionic segments of poly(methacrylic acid) (Figure 4(**6**)) and poly(sulfonic acid) (Figure 4(**7**)) are able to self-aggregate into phase separated ion-rich regions termed ionic clusters [176,177]. They are also capable of complexing with divalent counter ions, such as an organic diammonium, to drive the organization into complex structures, such as one-dimensional striped worms [178]. This electrostatic complexing approach to self-assembly can be extended to inorganic nanoparticles, coated with monovalent counter ions, such as primary amine-coated gold nanoparticles [178]. Besides, such polyanionic blocks are also able to organize into stable complexes with polycationic blocks, such as poly(dimethylbenzylammonium) [179] or poly(2-vinylpyridinium) (Figure 4(**8**)) [180,181], and originate a great variety of morphologies, such as core-shell micelles or multilayered membranes, exhibiting a striking *pH*- and solvent polarity-sensitivity.

#### 4.3.4. Mesogenic Segments

Mesogenic units are molecular fragments possessing a marked shape anisotropy, such as rod-like or disc-like motifs, that can self-organize into partially ordered mesophases, while retaining fluidity [182]. Their organization is triggered by several external stimuli, most notably temperature, uniaxial shearing/stretching, and electric and magnetic fields. In a polymer segment, the mesogenic units are incorporated either along the backbone (main-chain liquid crystals, Figure 4(**9**)) [183], or in side groups (side-chain liquid crystals, Figure 4(**10**)) [184]. The inclusion of a mesogenic segment is expected to impart its switchability in response to external stimuli to the whole polymer. In the case of side-chain mesogenic units, the length of the spacer connecting them to the backbone and their position, end-on versus side-on, are key factors that determine the resulting organization [185]. 

#### 4.3.5. Segments Containing Complementary Moieties

Besides the rather general affinities discussed above, there are also very specific affinities between the particular motifs, termed here as ligand–receptor pairs. When incorporated in different polymeric segments, they give rise to interactions based on molecular recognition, characterized by high directionality, a predefined stoichiometry, and large binding constants coupled with some degree of reversibility. An illustrative example is the interaction between polymeric segments possessing cyclodextrin (CD) side moieties and segments with aromatic residues acting as guest moieties (Figure 4(**11**)) [186]. In this case, not only the type of CD used (*α*-, *β*-, or *γ*-CD), but also the chemistry of the linkage between the side-moieties and polymeric backbone (e.g., amide versus ester linkage) play crucial roles on the self-organization behavior. Another example are the blocks incorporating boronic acid ester moieties (i.e., boronates) that possess a unique ability to form reversible covalent linkages to diols (e.g., sugars) [187,188], yielding cyclic boronate esters that are *pH*-responsive [189] and are therefore being considered for glucose sensing or *pH*-responsive delivery applications. Blocks including boronates in side groups (Figure 4(**12**)) are able to bind to segments containing saccharides or diol moieties in general. A related class of structural motifs are self-complementary moieties such as ureidopyrimidinone (Figure 4(**13**)). When present in side chain of different polymeric segments, they are able to form strong, but thermally reversible associations through complementary multiple hydrogen bonding [190,191].

A special case of ligand–receptor interactions in polymers are affinities toward single elements, in particular, philicities involving metallic elements. For example, some functional groups show a specific binding affinity to gold and their presence in a polymeric segment renders it aurophilic. Such groups afford a route to attach otherwise incompatible polymeric segments to gold surfaces, including Au-nanoparticles [199]. Particularly, thiol end-groups have been traditionally used to adsorb relatively thick polymeric layers onto gold, including proteins attached through cysteine residues [200], or to decorate the surface with initiator moieties from which a polymer layer is grown [198]. In a similar way, the high binding affinity of the primary amino groups (-NH_2_) to noble metals, including gold and silver, enable polymeric segments containing primary amine moieties, such as polyacrylamide, to act as a multiple anchor to gold surfaces. The other way around, gold clusters or nanoparticles can also be firmly bound to a polymeric surface [201]

## 5. Conclusions and Outlook

This review tries, for the first time, to give a systematic approach to the concept of philicities in polymer science. In addition to the already discussed philicities, there are many other possibilities. Thiophilic polymers are known and have been used since 1985 for the purification of immunoglobulins [202,203,204,205,206,207]. Also, polymers having cysteine repeat units can be considered as aurophilic, because of the strong affinity of SH-groups to gold [208]. It should be noted that the term aurophilicity has a different meaning in organic chemistry [209]. There can be a similar consideration for the term argentophilic. The introduction of boron-containing groups into the polymer structure leads to further possible applications [187,210,211,212,213,214], such as optical, electronic, and sensory [211], in medicine as sensors [187,212,213] or substrates [213]. Here, a certain philicity is given between boronic acid derivatives and diols. In relation to the interaction and self-structuring of amphiphilic polymers, the influence of the equilibrium of ionophobic and ionophilic groups was discussed [215,216,217,218,219,220]. A possible field of application for this purpose is the ion conductivity in polyelectrolytes [217,218,219]. Even liquid crystals show special philicities [221,222], depending on the structural blocks [221] and their shape [223,224]. Not only the chemical nature of a polymer, but also its microscopic morphology, can have a remarkable effect in its solvent-affinitity. For example, not only hydrophobic but also superhydrophobic surfaces can be generated by the selection of a strongly hydrophobic polymer in combination with sophisticated surface structuring [225]. Moreover, *omniphobic* surfaces, that is, the ability to repel not only water, but also liquids possessing a far lower surface tension than water, such as alkanes and alcohols, have been formed by the combination of particular re-entrant polymeric surface textures and long fluorinated chains [226,227].

The list of philicities/phobicities could be extended with many examples, and especially the list of elementophilicities would be very long. Several of these terms are used differently in organic chemistry and polymer science. It is expected that more and more polyphilic polymer architectures will be synthesized as a result of the ever increasing synthetic procedures towards precision polymers. This allows for a tailored incorporation of moieties with distinct affinities in complex environments. In future, to derive a comprehensive picture of philicities in all fields of chemistry, it would need a systematic approach of scientists.

## Figures and Tables

**Table 1:** Representative structure motifs present in polyphilic polymers (in addition to hydrophilic/lipophilic segments).

Affinity	Class	Segment/Block Example	Refs.
**Metallophilic**	Main-chain metal centers	Polymetallaynes: Ni, Pd, Pt, and	[166]
		Poly(ferrocenylsilane)s	[167,168]
		Gold-carboxylate	[170]
	Side-chain metal centers	Cobalt hexacarbonyl and Iridium phenylpyridine	[171,172]
	Supramolecular coordination	Supramolecular metal complexes with polymers incorporating nitrogen-containing ligands	[169,192,193]
**Ionophilic**	Electrolytes/ionomers	Poly(acrylic acid) complexes with diamines,	[178]
		Poly(methacrylic acid) complexes with aromatic polyamines (poly[2-vinylpyridinium]),	[180]
		Poly(sulfonic acid) clusters,	[177]
		Poly(sulfonic acid)s complexes with polycations (poly[dimethylbenzylammonium]),	[179]
		or aromatic polyamines [poly(2-vinylpyridinium)]	[181]
**Fluorophilic**	Fluorinated	Fluoroalkyl segments	[94,183,194]
**Siliphilic**	Carbosilanes	Poly(1,1-dialkylsilacyclobutane)s	[160,161]
	Siloxanes	Tris(trimethylsiloxy)silyl moieties	[159]
**Mesogenic**	Liquid crystals	Main-/side-chain liquid crystalline blocks	[182,183,184,185]
**Specific molecular recognition**	Ligand-receptor	Cyclodextrin-aromatics/or -adamantine	[186]
		Biotin-avidin	[195]
		Boronic acid-saccharides/or -polyols	[187,188,189]
		Thiol–gold nanoparticles	[196,197,198]
	Self-complementary	Ureidopyrimidinone	[190,191]
